# Generalize the use of the kidney failure risk equation (KFRE) for better vascular access planning

**DOI:** 10.1093/ckj/sfae060

**Published:** 2024-03-09

**Authors:** Bernardo Marques da Silva, Joana Gameiro

**Affiliations:** ULS Santa Maria, Department of Nephrology and Renal Transplantation, Lisbon, Portugal; Faculty of Medicine of the University of Lisbon, Lisbon, Portugal; ULS Santa Maria, Department of Nephrology and Renal Transplantation, Lisbon, Portugal; Faculty of Medicine of the University of Lisbon, Lisbon, Portugal

To the Editor,

We were pleased to read the work by Atiquzzaman *et al.* concerning vascular access (VA) planning and the possibility of improvement by using the kidney failure risk equation (KFRE) [1]. Given the rising prevalence of chronic kidney disease (CKD) and the increasing number of patients requiring hemodialysis (HD), an effective VA [arteriovenous fistula or graft (AVF/G)] at the start of HD is crucial in ensuring optimal patient outcomes and quality of life [2]. Ideally, patients should be referred not only for VA creation in a timely manner, but also to prevent creation in patients who will not require HD. Referral criteria for AVF/G creation are still insufficient and rely on the nephrologist's perception of when HD will be required [3]. Therefore, developing tools to improve VA planning and timely creation is of utter importance.

In this recently published population-based study of 2581 participants with median age of 71 years, median eGFR at index 14.0 mL/min/1.73 m^2^, and 2-year KFRE of 48.9%, 61% of patients started HD within 2 years. Among those, 43% had AVF/G at HD start and had a significantly higher KFRE score (60 vs 29%, *P *< 0.001). More importantly, the authors demonstrated that using a KFRE cut-off >40% in addition to eGFR <20 mL/min/1.73 m^2^ significantly improved the adequateness of VA creation. Indeed, the proportion of patients who started HD on AVF/G within 2 years was 49% for eGFR threshold-based referral and of 58% when an adjunct KFRE of >40% was used. Despite the high sensitivity of this cut-off, the low specificity resulted in the lack of referral for AVF/G creation in 366 patients who started HD within 2 years.

Our retrospective study of 256 patients had a similar age and eGFR at referral for VA creation, and the mean 2-year KFRE score was 30.4% [4]. In a 2-year follow-up, 62.1% started HD. In this population, the optimal KFRE cut-off for VA referral was ⩾20%, which had a sensitivity of 72.8% and specificity of 78.4%. Eighty-four percent of patients with a KFRE ⩾20% started HD within 2 years. However, this was a retrospective cohort and would require external validation for a generalized use.

**Figure 1:**
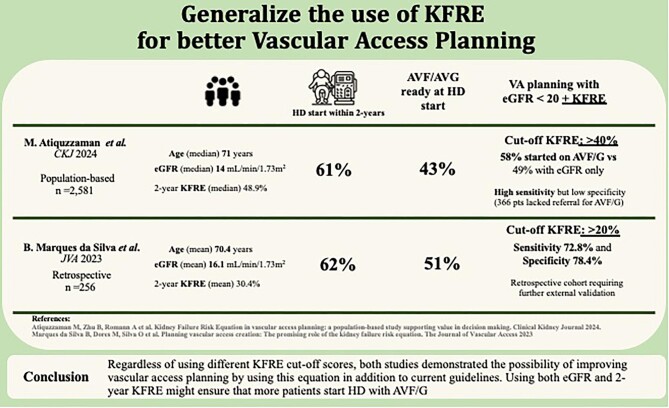
A visual summary of studies using KFRE for VA planning.

Regardless of using different KFRE cut-off scores, both studies demonstrated the possibility of improving VA planning by using this equation in addition to current guidelines, as described in Fig. 1. Using both eGFR and 2-year KFRE might ensure that more patients start HD with AVF/G and decrease unnecessary VA procedures. In an area so crucial for CKD patients, more studies should be undertaken to further improve and validate this additional tool in different population cohorts.
